# Acute small bowel obstruction: a rare initial presentation for the metastasis of the large-cell carcinoma of the lung

**DOI:** 10.1186/1477-7819-10-26

**Published:** 2012-01-29

**Authors:** Yongmao Song, Modan Li, Jianzhen Shan, Xiaoxian Ye, Shangyi Tang, Xuefeng Fang, Kefeng Ding, Ying Yuan

**Affiliations:** 1 Department of Medical Oncology, the 2nd Hospital of Zhejiang University College of Medicine, Hangzhou, Zhejiang, 310009, P.R. China; 2 Department of Surgical Oncology, the 2nd Hospital of Zhejiang University College of Medicine, Hangzhou, Zhejiang, 310009, P.R. China

**Keywords:** lung cancer, metastasis, obstruction, gastrointestine

## Abstract

We present one case with symptom of paroxysmal abdominal pain for over 20 days. Abdominal computerized tomography (CT) scan revealed intestinal obstruction and a mass of 6.0 cm × 6.0 cm in size located at the left adrenal. Chest CT scan showed a lobulated mass of 2.7 cm × 2.7 cm in size at the upper left lung. Core needle biopsy of the lung mass confirmed the diagnosis of large cell carcinoma. The patient underwent an emergency abdominal laparotomy and received a chemotherapy regimen that consisted of pemetrexed and cisplatin postoperatively. In addition, we made a review of the literature of the occurrence, diagnosis and outcome of this manifestation.

## Background

Primary lung cancer is one of the leading causes of death in China. Gastrointestinal (GI) metastasis as the initial presentation of primary lung cancer is relatively rare in the clinic. Acute GI symptoms such as obstruction, perforation or bleeding could be the results of small bowel involvement. We report one case of metastatic large cell carcinoma of the lung with the primary presentation of acute small bowel obstruction. The current management plan, as well as prognosis of this patient is discussed. GI metastasis of lung cancer occurs late in the course of the disease and is associated with serious clinical complications and outcomes. Also, we made a review of the literature relevant to the occurrence, diagnosis and outcome of GI metastases of lung cancer.

## Case presentation

**Figure 1:**
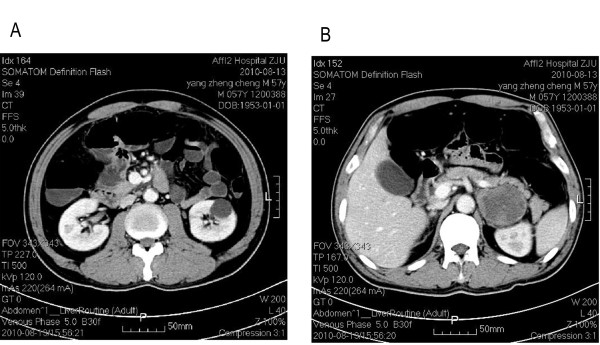
**The abdominal computerized tomography scan revealed intestinal obstruction (a) and a mass measuring of 6.0 cm × 6.0 cm in size which showed enhancement in intensified tomography near the left adrenal gland (b)**.

**Figure 2:**
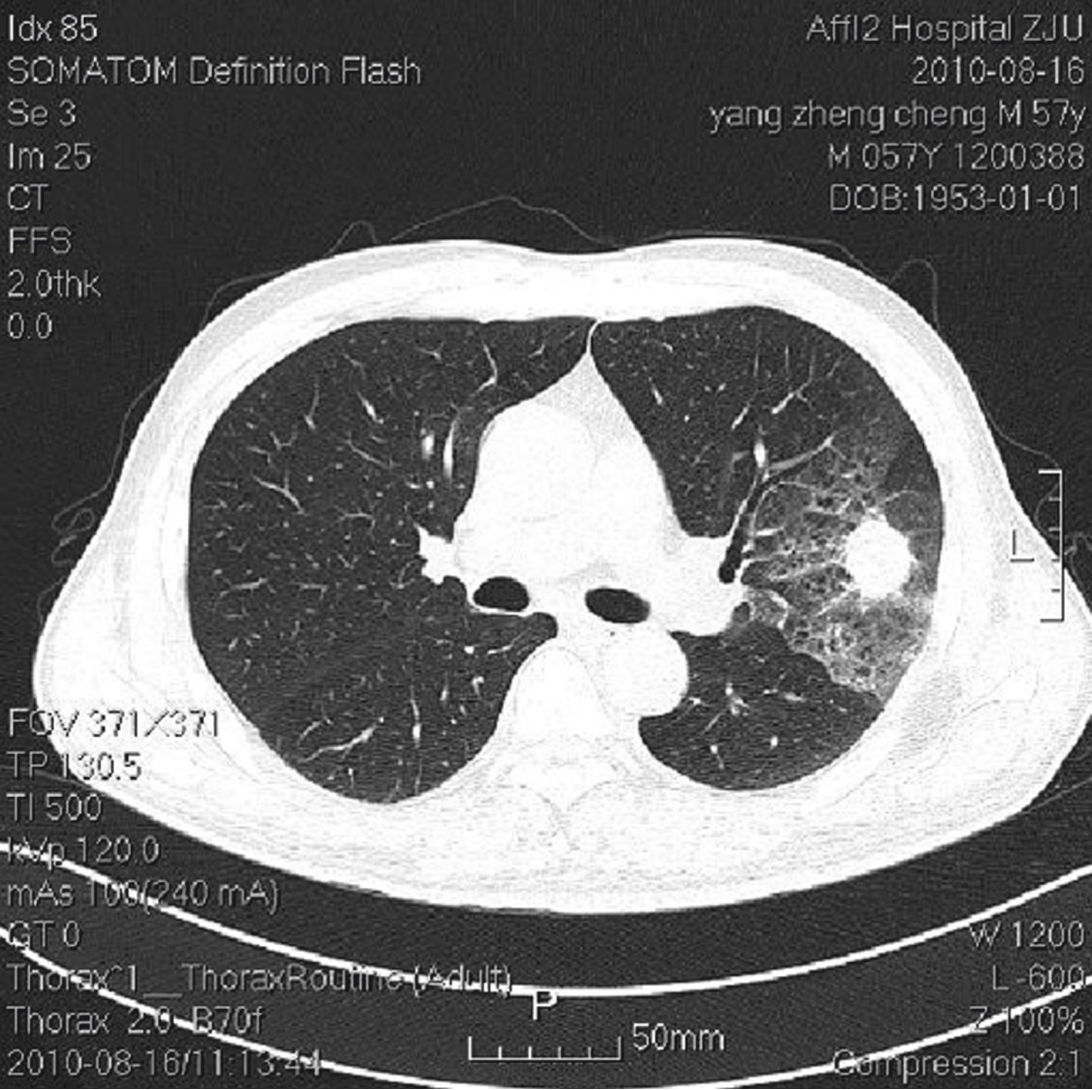
**The chest computerized tomography scan showed a 2.7 cm × 2.7 cm lobulated mass at the upper left lung with localized pneumonia changes**.

**Figure 3:**
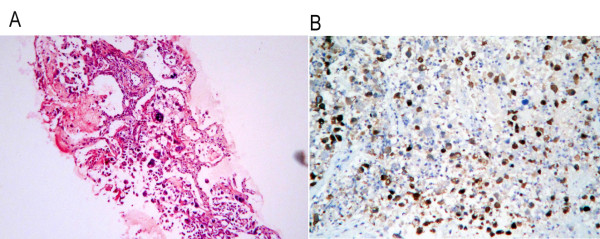
**Microscopic images of lung biopsy (a) hematoxylin-eosin staining × 100, showing large, undifferentiated tumor cells with rhabdoid variant; (b) immunoperoxidase × 400 showing CK7 immunoreactivity of cancer cells**.

A 58-year-old male patient with a history of meningioma surgery was admitted to the Emergency Department with a chief complaint of episodes of paroxysmal abdominal pain. The pain started 20 days previously that was aggravated for the last 2 days. Slight tenderness over the left epigastric region and active bowel sounds were observed during physical examination. Plain abdominal X-ray showed dilatation of small bowel with multiple gas-fluid levels. The abdominal computerized tomography (CT) (Figure 1) scan revealed intestinal obstruction and a mass of 6.0 cm × 6.0 cm in size which showed enhancement in contrast-enhanced CT near the left adrenal gland. The chest CT (Figure 2) scan showed a 2.7 cm × 2.7 cm lobulated mass at the upper left lung with localized pneumonia changes. Brain MRI suggested no evidence of cerebral or cerebellar metastasis. A CT-guided core needle biopsy of the lung mass confirmed the diagnosis of large cell carcinoma of the lung(Figure 3).

**Figure 4:**
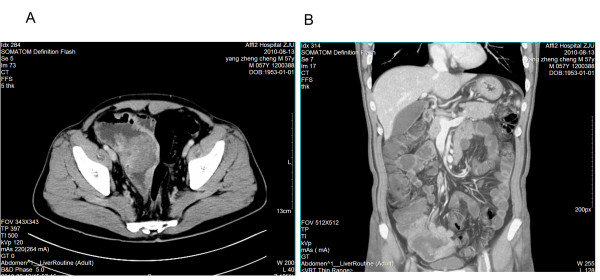
**The abdominal computerized tomography scan revealed metastatic tumor mass of jejunum (a and b)**.

**Figure 5:**
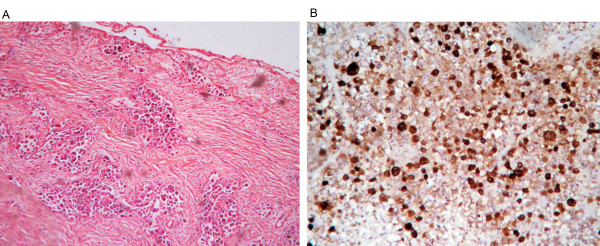
**Microscopic images of jejunum resection (a) hematoxylin-eosin staining, × 100 showing groups of large anaplastic tumor cells invading the lamina serosa; (b) immunoperoxidase × 400 showing CK7 cytoplasmic positivity on a jejunum resection tissue**.

Due to the life-threatening nature of complete obstruction, the patient underwent an emergency abdominal laparotomy. Exploration of the abdominal cavity revealed a mass of 12.0 cm × 10.0 cm in size in the jejunum which had already invaded the bladder. The abdominal computerized tomography scan revealed metastatic tumor mass of jejunum (Figure 4). The mass, the invaded jejunum and part of the bladder was resected. Another hard and fixed mass of 6.0 cm × 6.0 cm in size near the left adrenal gland was also identified and it was not removed during the procedure due to its invasion to the surrounding of great vessels. Post-operative pathological analysis of the surgical specimen showed the presence of poorly differentiated carcinoma with rhabdoid differentiation (Figure 5). Immunohistochemical (IHC) staining revealed that the tumor cells were positive for CK7, vimentin, and partially positive for CA153 receptors. Combining with the results of the lung biopsy, we concluded that the jejunal mass was a metastasis from the primary large cell carcinoma of lung.

Postoperatively, the patient complained severe continuous upper left abdominal pain which intensified progressively. Additional CT scan revealed that the mass near the left adrenal gland had swollen to 8.8 cm × 8.3 cm in size, while the upper left lung mass had increased to 4.5 cm × 4.0 cm in size, and localized pneumonia, atelectasis and pleural effusion of left thoracic cavity had also developed. The patient was diagnosed with stage IV large cell lung cancer with jejunum and left adrenal gland metastasis. The chemotherapy regimen consisted of pemetrexed 500 mg/m^2 ^at day 1 and cisplatin 25 mg/m^2 ^on day 1 to day 3. However, the abdominal pain was not relieved with this treatment. Two weeks after the first cycle of chemotherapy, the patient suddenly presented with abnormal activities of the right limbs, with a positive result in the finger-to-nose test. New brain MRI scan revealed multiple metastatic lesions with prominent surrounding edema in various cerebral and cerebellar regions. The patient suffered an epileptic seizure and died three days later.

## Discussion

The most frequently involved metastatic organs of lung cancer are bones, liver, brain and adrenals, while metastases to the gastrointestinal system is relatively rare with a frequency ranging from 0.2% to 1.7% in several studies [1-3]. However, autopsy data have suggested a much higher frequency of GI metastases of lung cancer than clinically reported cases and there is a prevalence of 4.7% to 14% [4,5]. Small bowel is the most commonly reported GI metastatic site of lung cancer [5,6]. Clinically significant metastases to the small bowel are rare and occur only in the advanced stage of lung cancer. Most patients with small bowel metastases have no specific symptoms such as anorexia, abdominal pain, distention and diarrhea. With the progression of disease, life threatening symptoms do present, such as small bowel obstruction, perforation or even bleeding[7-10]. Hillenbrand reviewed the literature from 1967 to 2003 and found 58 documented cases with metastasis to the small bowel of primary lung cancer. Over 80% of the cases with small bowel metastases were male, with ages ranging from 36 to 78 years old and metastases presented as perforation (59%), obstruction (29%) and hemorrhage (10%) [11]. In addition, McNeill *et al*. [6] reported that small bowel metastases were always associated with other metastatic sites (with an average of 4.8 sites), thus suggesting a late stage of the lung cancer. These patients often had a poor prognosis with a life-expectancy less than 16 weeks.

Yang *et al*. [1] reported in their study that squamous cell carcinoma is the most common cell type of lung cancer which develop GI metastases [2,4,12]. But other studies showed that poorly differentiated adenocarcinoma and large cell carcinoma of the lung have higher incidence of GI metastases. McNeill *et al*. [6] reported that 12 of 31 (39.0%) patients with large cell carcinoma had small bowel metastases. Yoshimoto *et al*. [4] evaluated 470 cases of GI metastases from primary lung cancer over 33 years and their data showed that 30% of cases were large cell carcinoma. Patients with large cell carcinoma had a significantly higher rate of GI metastases (P = 0.004, odds ratio 3.524) compared with patients with non-large cell carcinoma. Because of the difficulty in early detection of GI diseases, the diagnosis of small bowel metastases was often delayed before it presented with life-threatening complications, which frequently required emergency surgeries [1,13]. Kim evaluated the CT scan findings in 28 patients with gastrointestinal metastasis from lung cancer and found 5/26 patients had two lesions and 21 patients had only one lesion. The shape of GI lesion varied on CT scans, presenting as wall thickening in 14 cases, an intraluminal polypoid mass in 14 cases, and an exophytic mass in the other three cases [14]. PET is more accurate than CT or other conventional imaging methods for the diagnosis of metastatic malignant sites. However, the role of PET in the diagnosis of lung cancer GI metastasis is still controversial because of the lack of enough clinical cases. Small bowel metastases from primary lung cancer were usually confirmed by pathological analysis, with the help of immunohistochemical staining of TTF-1, CDX2, CK7 and CK20, to differentiate the primary small bowel tumor from metastases of lung cancer [2].

So far, surgical resection is the mainstay treatment for small bowel metastases from large cell carcinoma of the lung. Individualized treatment may also be helpful. Advances in chemotherapy and supportive care may lead to the improvement of the survival rate for these lung cancer patients. According to the report from a phase III study (JMDB trial), overall survival was statistically higher in patients with large cell carcinoma histology who received treatment of cisplatin plus pemetrexed versus cisplatin plus gemcitabine, the latter being the standard first-line regimen of non-small cell lung cancer; n = 153; 10.4 months v 6.7 months, respectively [15].

In our case, small bowel obstruction was the initial clinical symptom for this male patient. Primary lung large cell carcinoma was confirmed by a pathologist and at the same time small bowel and left adrenal metastases were suggested by CT scan (stage IV). Unfortunately the patient did not benefit from an emergency operation and chemotherapy. Multiple brain metastases soon developed and the patient died 64 days after the diagnosis. The histological type of large cell carcinoma of the lung, metastases in multiple sites, heavy tumor load and surgical stroke might be the reasons of the poor prognosis. During the clinical course, physicians should be aware of gastrointestinal tract metastases if the patient with lung cancer presents with gastrointestinal tract symptoms. Progression of examinations for screening small bowel diseases will be helpful for doctors to diagnose more cases of small bowel metastases from lung cancer in the future. Earlier resection of the gastrointestinal metastases to alleviate the symptoms and decrease tumor load may be beneficial to the patients and achieve a better prognosis [11,16-18].

## Conclusion

In conclusion, small bowel metastases from lung cancer are not uncommon and occur only in the advanced stage of lung cancer, particularly if patients have the histological type of large cell lung carcinoma. Physicians should keep in mind that the incidence of small bowel metastases as a site of lung cancer spread is increasing as patients with lung cancer now live longer due to improved treatment.

## Consent

Written informed consent was obtained from the patient for publication of this case report and accompanying images. A copy of the written consent is available for review by the Editor-in-Chief of this journal.

## Abbreviations

CT: computerized tomography; GI: gastrointestinal; IHC: immunohistochemical.

## Competing interests

The authors declare that they have no competing interests.

## Authors' contributions

YM S wrote the manuscript. YM S, KF D and SY T performed surgery. Y Y carried out the pathological examination. Y Y and XF F was involved in the final editing. Y Y, MD L, XX Y, JZ S carried out chemothreapy. All authors approved the final manuscript
